# Comprehensive Comparison of AAV Purification Methods: Iodixanol Gradient Centrifugation vs. Immuno-Affinity Chromatography

**DOI:** 10.1155/2023/2339702

**Published:** 2023-12-11

**Authors:** Anh K. Lam, Patrick L. Mulcrone, Dylan Frabutt, Junping Zhang, Matthew Chrzanowski, Sreevani Arisa, Maite Munoz, Xin Li, Moanaro Biswas, David Markusic, Roland W. Herzog, Weidong Xiao

**Affiliations:** 1 Department of Pediatrics, Herman B Wells Center for Pediatric Research, Indiana University School of Medicine, Indianapolis, IN 46202, USA; 2 Lewis Katz School of Medicine, Temple University, Philadelphia, PA 19140, USA

## Abstract

Recombinant adeno-associated viruses (AAVs) have emerged as a widely used gene delivery platform for both basic research and human gene therapy. To ensure and improve the safety profile of AAV vectors, substantial efforts have been dedicated to the vector production process development using suspension HEK293 cells. Here, we studied and compared two downstream purification methods, iodixanol gradient ultracentrifugation versus immuno-affinity chromatography (POROS^™^ CaptureSelect^™^ AAVX column). We tested multiple vector batches that were separately produced (including AAV5, AAV8, and AAV9 serotypes). To account for batch-to-batch variability, each batch was halved for subsequent purification by either iodixanol gradient centrifugation or affinity chromatography. In parallel, purified vectors were characterized, and transduction was compared both *in vitro* and *in vivo* in mice (using multiple transgenes: Gaussia luciferase, eGFP, and human factor IX). Each purification method was found to have its own advantages and disadvantages regarding purity, viral genome (vg) recovery, and relative empty particle content. Differences in transduction efficiency were found to reflect batch-to-batch variability rather than disparities between the two purification methods, which were similarly capable of yielding potent AAV vectors.

## 1. Introduction

Gene therapeutic agents are having a major, positive impact on researching and treating various diseases. Viral vector-based gene therapies are among the most efficient approaches for the correction of genetic defects. Among these, adeno-associated viruses (AAV) are most widely used for *in vivo* gene transfer, which is reflected in the five current FDA-approved AAV therapeutics for distinct genetic disorders: HEMGENIX [1] (AAV5, hemophilia B), ZOLGENSMA [2] (AAV9, spinal muscular atrophy), LUXTURNA [3] (AAV2, retinal dystrophy), ROCTAVIAN (AAV5, hemophilia A), and ELEVIDYS (AAVrh74, Duchenne muscular dystrophy). Towards clinical approval, translational studies and preclinical studies require a high concentration of AAV titers to enable testing of design specifications, efficacy, side effects, and outcomes of the AAV gene transfer. To achieve the large quantities of vector required for such studies, production is scaled up by growing and transfecting suitable cells, such as human embryonic kidney cells (HEK293), in roller bottles, through continuous perfusion, or in WAVE Bioreactors [4]. Naturally, these scale-up processes result in not only larger amounts of the desired AAV but also of possible contaminants such as cellular proteins, membrane components, nucleic acid fragments, and empty capsid AAV particles. Therefore, purification of the AAV drug product is essential prior to carrying out translational and efficacy studies.

The two most conventional methods for AAV purification are cesium chloride (CsCl) and iodixanol gradient ultracentrifugation (IOD), which separate particles based on their buoyant density or sedimentation rates. These methods are independent of capsid serotypes and allow the separation of empty and full virions. Because the CsCl-based method is time-consuming and may introduce toxicity, iodixanol has become a more preferred method [5]. For a more scalable process, column chromatography is well established and has been adapted for AAV purification [6, 7]. Immuno-affinity chromatography specifically utilizes an affinity-binding interaction between the column resin and the desired product to be purified. Clinical-grade AAV vectors have been reported to be purified using both methods of gradient centrifugation and column chromatography, or a combination of both, since the ultracentrifugation method is difficult to scale up [8–12]. Given the importance of the purification methodology (under the CMC Guidance for Industry from the US FDA) [13], we compared two commonly used purification methods across various AAV serotypes to determine if biological differences *in vitro* and *in vivo* could be affected by a specific purification approach.

**Figure 1:**
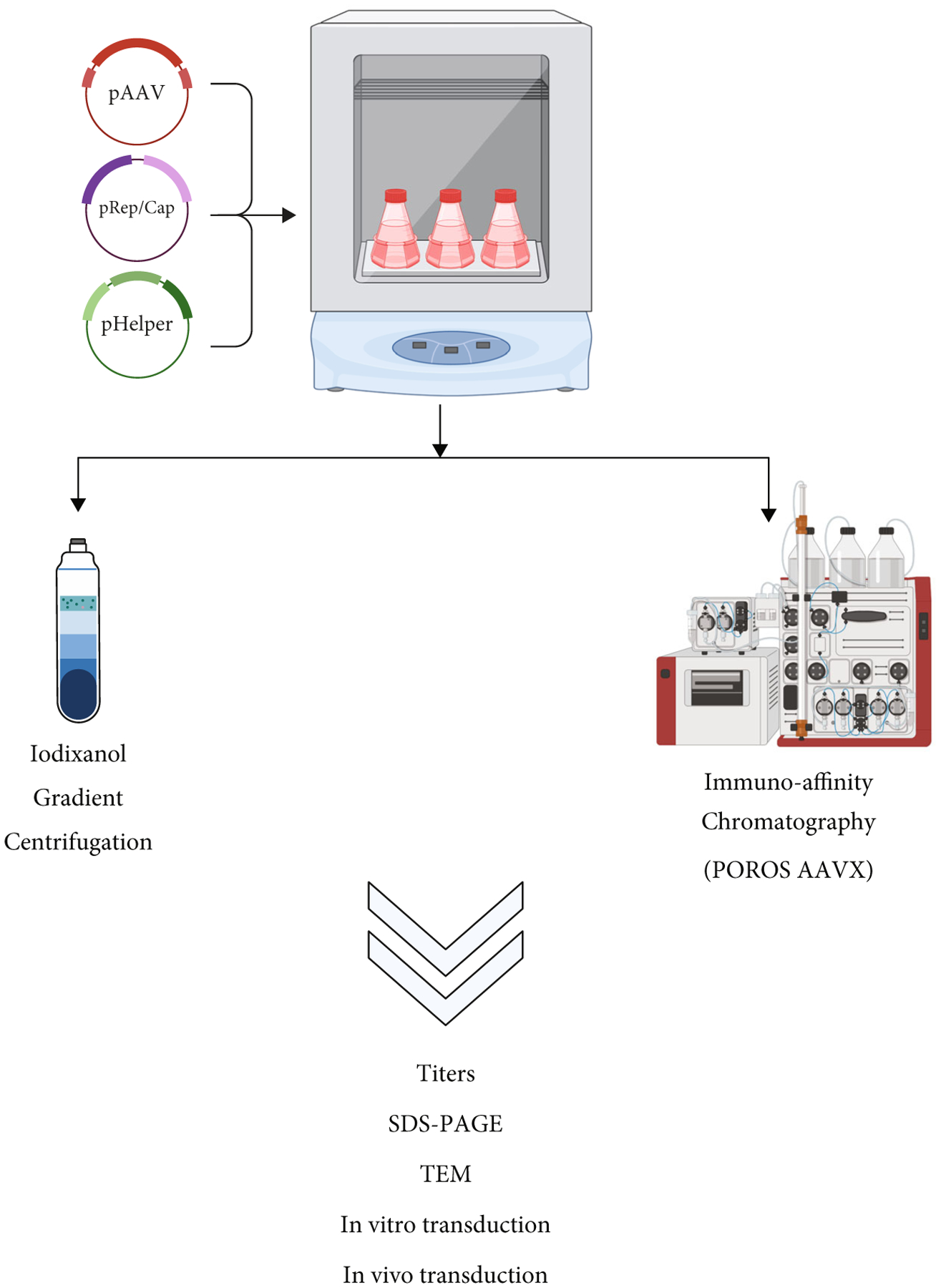
Schematic diagram of the AAV purification comparison study. Suspension HEK293 cells in orbital shake flasks were used to produce recombinant AAVs by the triple-plasmid transfection method. Each batch was split in half for subsequent purification by either iodixanol gradient centrifugation or immune-affinity chromatography (POROS^™^ CaptureSelect^™^ AAVX column). The resulting vectors were then studied and characterized by both *in vitro* and *in vivo* assays.

To avoid overinterpretation and batch-to-batch errors, separate lots of AAV vectors were produced and analyzed independently. In this study, we report comprehensive comparison data of three independent AAV batches (AAV5, AAV8, and AAV9) purified by one round of either iodixanol gradient centrifugation (IOD) or affinity chromatography using the POROS^™^ CaptureSelect^™^ AAVX column (POR). These data include characterizations of purity, the relative presence of empty capsids (SDS-PAGE and transmission electron microscopy), and titers and plasmid backbone contaminations (qPCR). Furthermore, transduction efficiencies were determined *in vitro* and *in vivo* (using self-complementary AAV-CB-eGFP, AAV-CB-GLUC, and AAV-TTR-cohFIX constructs) as outlined in Figure 1.

## 2. Results

**Figure 2:**
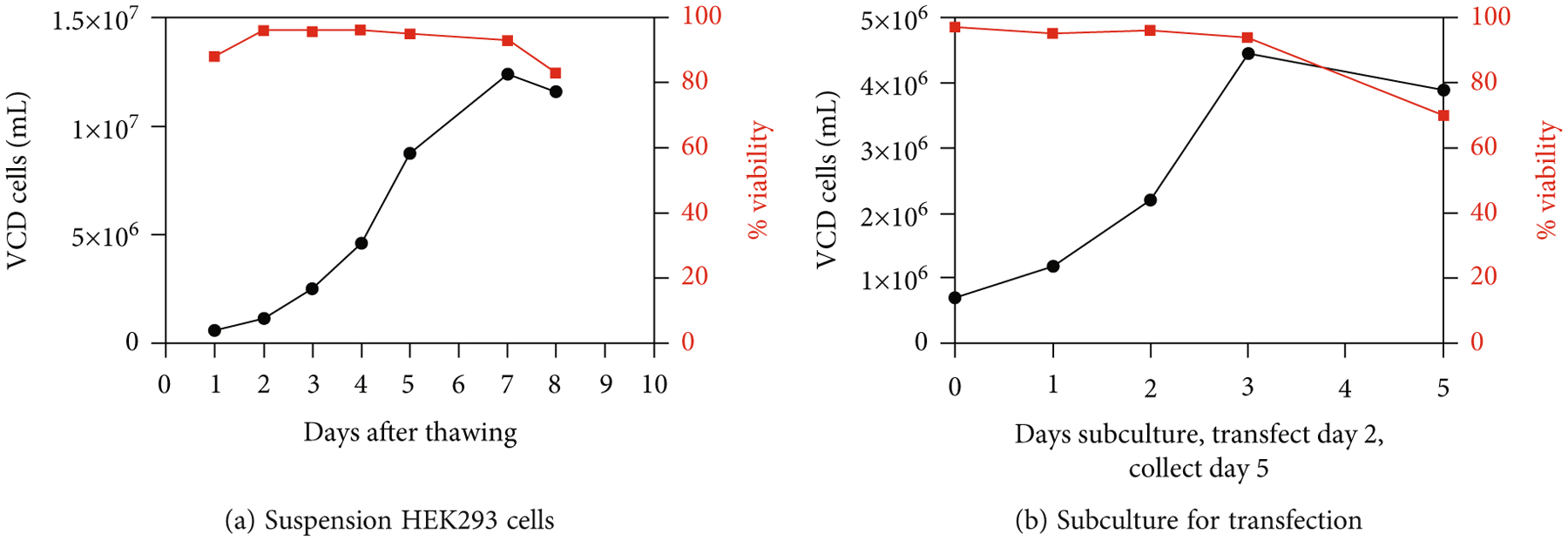
Viability and viable cell density (VCD) representation of scalable suspension system HEK293 cells: freshly thawed cells (a) and subcultured cells (b). Transfection was performed on day 2 with a VCD of ~2.0–2.5 × 10^6^ cells/mL and viability of >92% and vector harvest was at day 5, which was 72 hr posttransfection.

Orbital shake flasks for cell culture were used for a research scale of 125 mL-1000 mL (max 25% volume of cell culture). A suspension system of HEK293 cells was optimized so that on the day of transfection, the viable cell density (VCD) and % viability were ~2.0 to 2.5 × 10^6^ cells/mL and >92%, respectively (Figure 2).

At the time of vector harvest, 72 hrs posttransfection, the cells and media were collected and split in half for parallel purification by IOD or POR. For downstream analyses, purified vectors from both methods were side-by-side characterized, tittered, and transduced cells *in vitro* and IV-injected via the tail vein of mice for transgene expression comparison.

**Figure 3:**
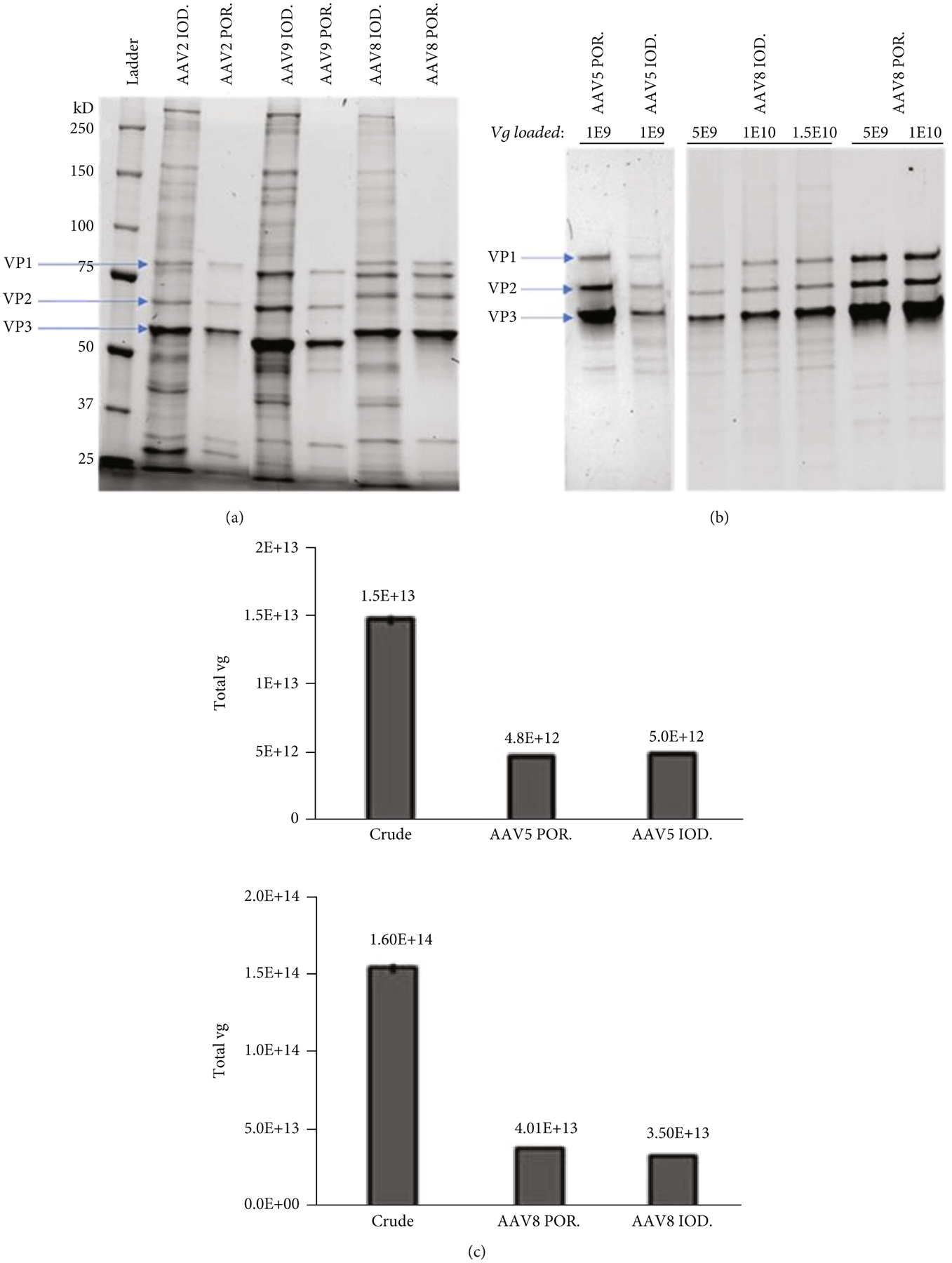

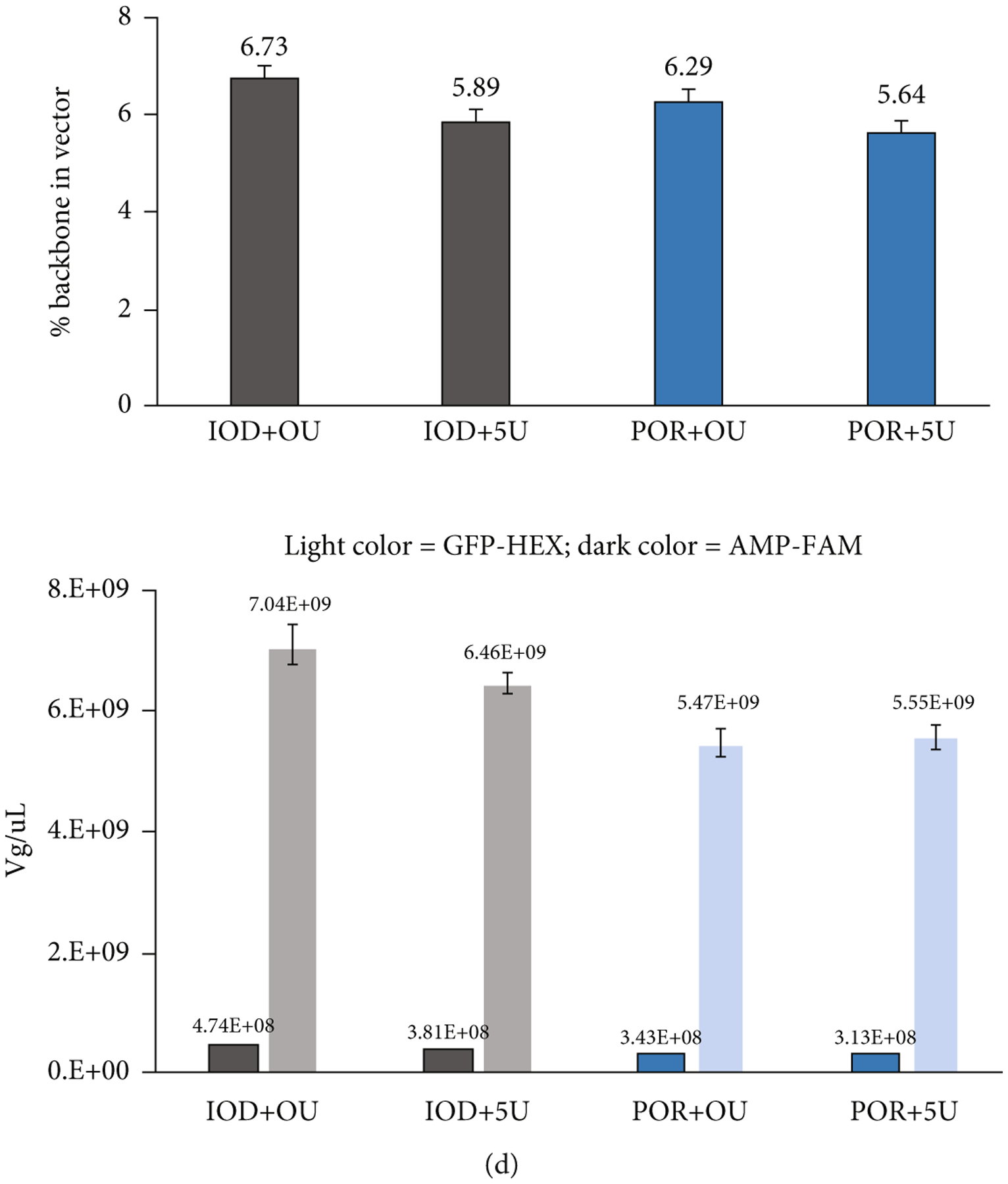
AAV vector genome and capsid protein characterization using SDS-PAGE (7.5% gel) and qPCR probe-based titration. POROS-AAVX affinity column purified (POR) vectors are purer than their iodixanol (IOD) purified counterparts (a). With the same titer of vg loaded, POR vectors show darker bands which indicate more empty capsids than IOD vectors (b). Using qPCR probe-based analysis, vg recovered by both purification methods are comparably similar ((c) top: from 50 mL of triple-plasmid-transfected cell culture; bottom: from 200 mL). Backbone contaminations were determined and multiplexed using an ampicillin-primers/probe, which indicated a similar amount of the plasmid backbone packaged by AAV8 purified by both methods (d). 0 U = no pretreatment of DNase I before qPCR titration; 5 U = 5 units of DNase I pretreatment were used prior to qPCR.

Multiple batches of AAVs including AAV5, AAV8, and AAV9 were characterized by SDS-PAGE to compare their relative purity. As shown in Figures 3(a) and 3(b), AAV vectors generated via the IOD method were often less pure and contaminated with other proteins, as detected by smears in addition to the three bands corresponding to VP1, 2, and 3. When normalized to the vg loaded per lane of SDS-PAGE, the POR method yielded approximately 2- to 5-fold more empty capsids compared to the IOD method, as indicated by the darker bands for VP1, 2, and 3 (Figure 3(b), estimate based on pixel contrast by ImageJ).

The titers of AAV vg recovered by both purification methods were found to be comparatively similar (Figure 3(c)). Plasmid backbone contamination was also examined by using primers and probes that target ampicillin. As shown in Figure 3(d), pretreatment of DNase I prior to vg titration reduced the % of plasmid backbone contamination in both IOD and POR samples from ~6.5% to ~5.7%, which could be from impurity DNA/plasmid fragments stuck on the capsids.

**Figure 4:**
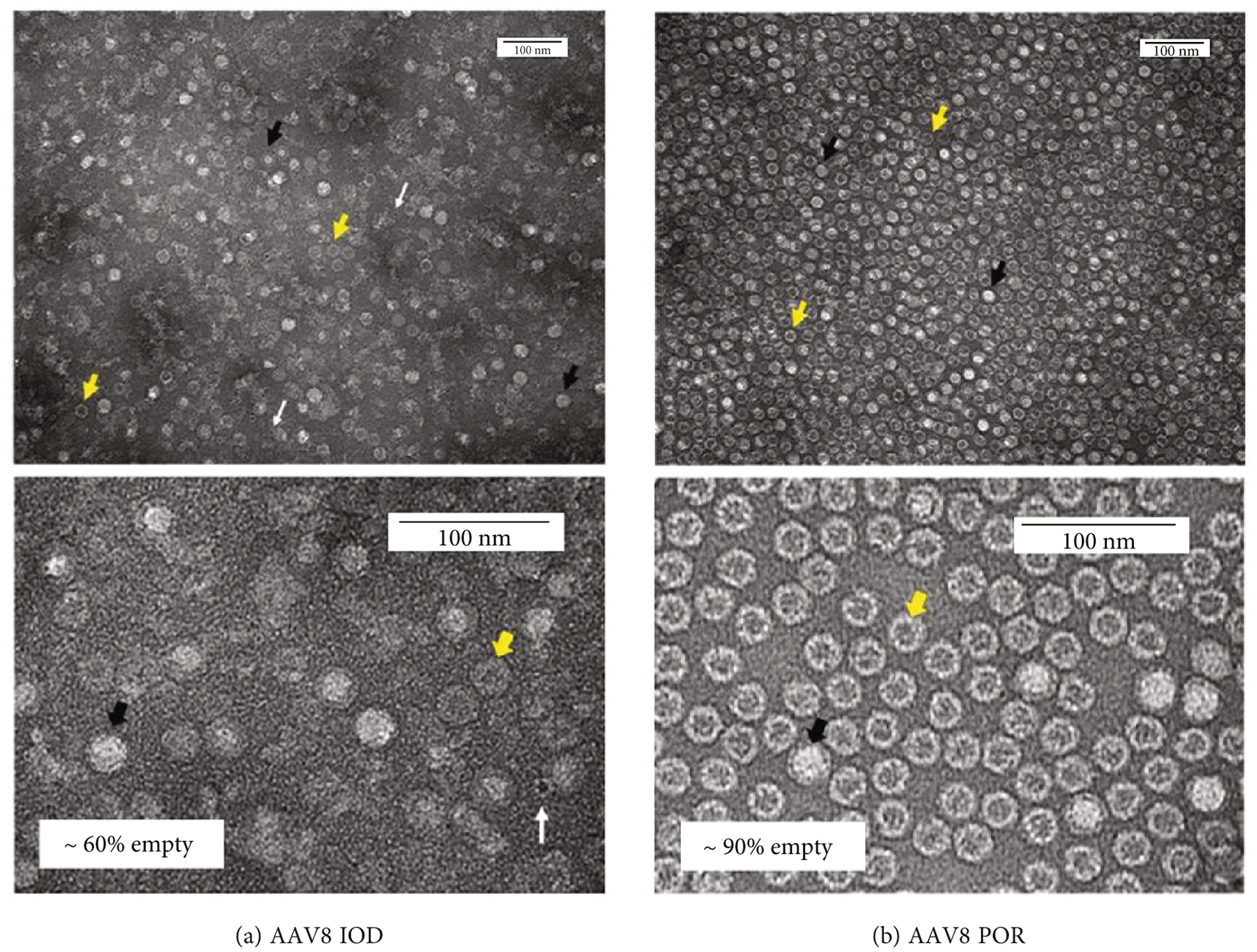
Transmission electron micrographs of AAV8 purified by iodixanol gradient centrifugation (a) or by POROS AAVX column (b). Both samples were imaged at 5E9 vg/*μ*L with a negative stain. More impurities were identified from iodixanol gradient centrifugation (a) including ferritin, as indicated by white arrows, as identified by previous studies [14]. Empty capsids—indicated by yellow arrows—were observed as denser virions from affinity column purified vectors (b). The empty capsid amount was estimated and shown on the lower panels. Black arrows indicate full capsids; scale bars = 100 nm.

Images from TEM confirmed more impurities in IOD vectors as many patches of debris are observed surrounding each virion, leading to poorer image quality (Figure 4(a)). One common impurity from the IOD purified vector appears as smaller circles (indicated by white arrows) which was identified by previous studies to be ferritin [14]. Greater purity was found in POR-purified vectors, but more empty capsids (80–90%, based on ImageJ counting of ~1000 particles) were seen in POR vectors (TEM images in Figure 4). This confirms the data from SDS-PAGE in Figure 3.

**Figure 5:**
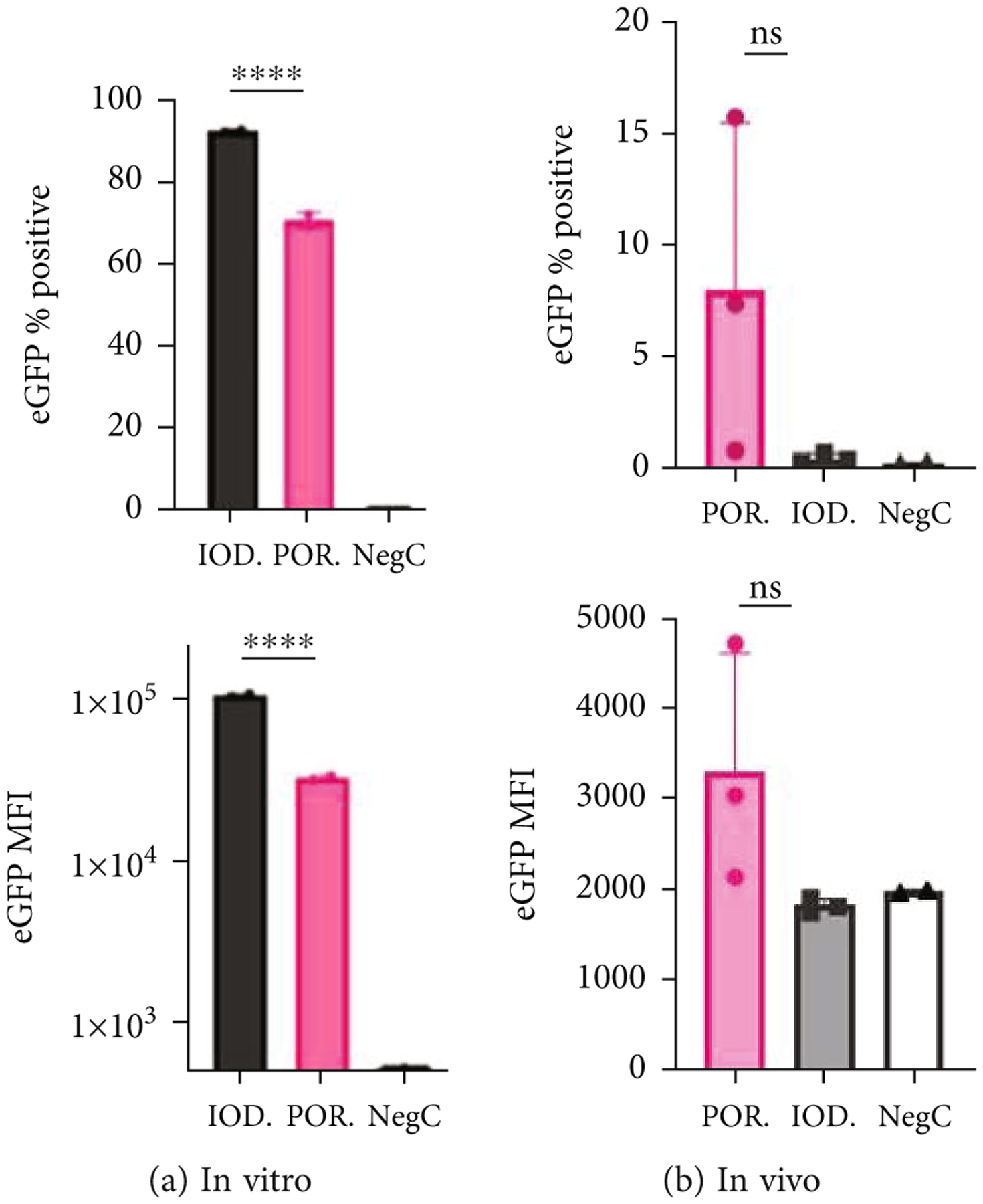
Transduction efficiency of AAV5-CB-eGFP measured by flow cytometry. *In vitro* transduction results of GM16095 cells ((a) MOI = 100 K; *n* = 2 wells/group). *In vivo* transduction results of WT BALB/c mouse livers at week 2 post-IV-injection ((b) *n* = 3/group; dose = 1 × 10^11^ vg/mouse). A two-way ANOVA with Turkey’s multiple comparisons was performed. MFI = mean fluorescence intensity; %eGFP positive = percent positive cells of eGFP. *P < 0.05, **P < 0.01, ***P < 0.001, ****P < 0.0001.

Transduction efficiency was determined *in vitro* by infecting human GM16095 fibroblast cells and quantifying gene expression after 48–72 hrs of AAV infection. Figure 5(a) shows transduction efficiency by AAV5-CB-eGFP measured by flow cytometry as % GFP-positive cells and mean fluorescent intensity (MFI) signals. Despite the difference in empty capsids per group, the multiplicity of infection (MOI) was normalized based on vg content titrated by qPCR. At the same MOI of 100,000, IOD-purified AAV5 had significantly higher transduction efficiency than POR AAV5 (~90% eGFP >70% eGFP positive), which indicated that the excess number of empty capsids detrimentally affects the transduction in vitro. However, *in vivo* testing demonstrated opposite results, as POR AAV5 trended to transduce the livers of WT BALB/c mice better than IOD AAV5, although this difference did not reach statistical significance (Figure 5(b)).

**Figure 6:**
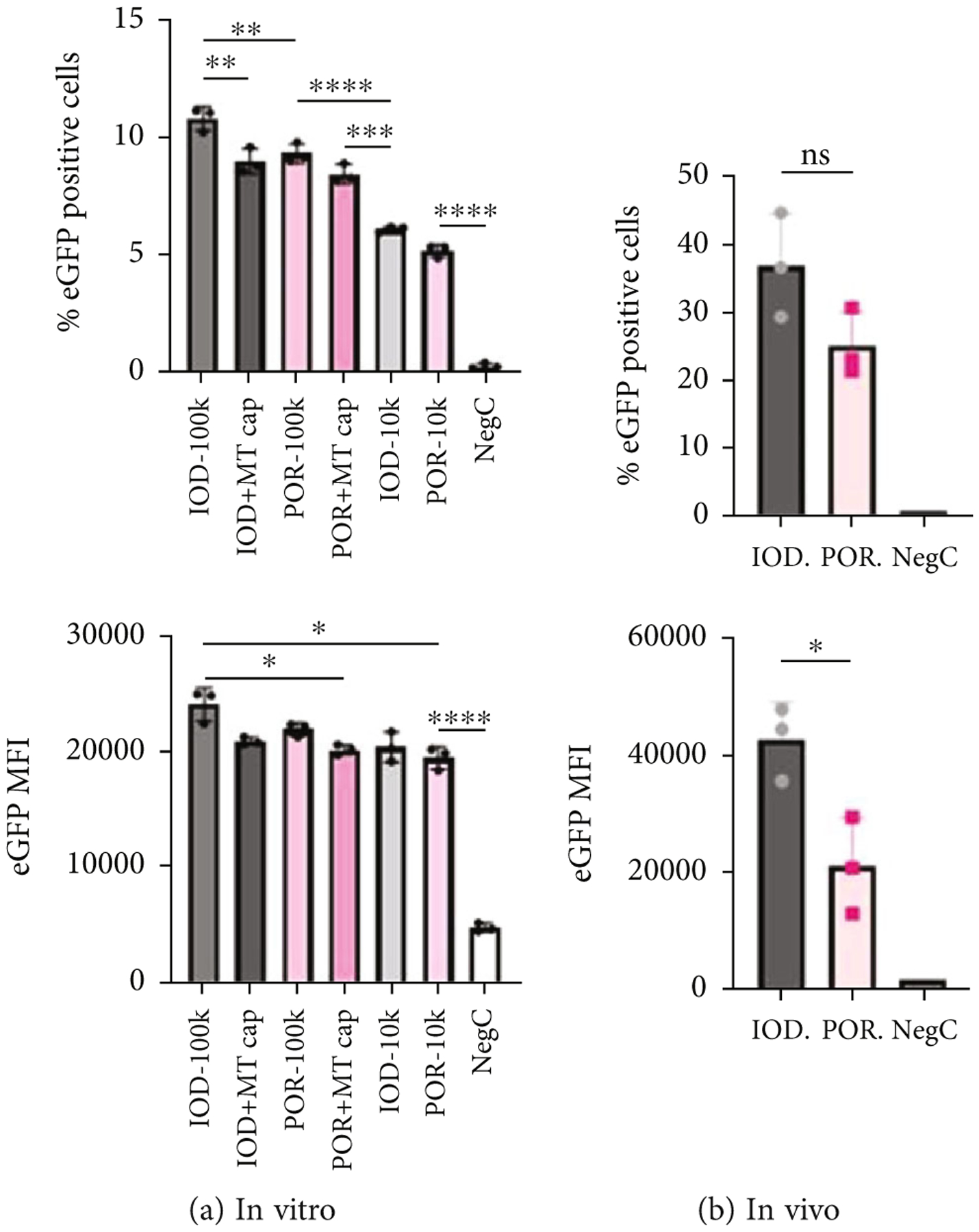
Transduction efficiency of AAV8-CB-eGFP measured by flow cytometry. *In vitro* transduction results of GM16095 cells ((a) MOI = 100 K or 10 K; *n* = 3 wells/group; MT cap = empty capsids). *In vivo* transduction results of WT BALB/c mouse livers at week 2 post-IV-injection ((b) *n* = 3/group; dose = 2 × 10^11^ vg/mouse). A two-way ANOVA with Turkey’s multiple comparisons was performed. MFI = mean fluorescence intensity; %eGFP positive = percent positive cells of eGFP. *P < 0.05, **P < 0.01, ***P < 0.001, ****P < 0.0001.

In another study using AAV8-CB-eGFP, an *in vitro* transduction assay showed the same trend, as IOD AAV8 outperformed POR AAV8 at the same MOI in GM16095 cells (Figure 6(a)). To determine the effect of empty capsids, we added an equivalent of virions (based on SDS-PAGE) without any packaged transgene to the same MOI of IOD AAV8 or POR AAV8; as a result, the transduced cells measured as % GFP positive were significantly decreased by the addition of empty capsids (Figure 6(a)). This indicated that empty capsids, *in vitro*, could potentially compete for the AAV trafficking machinery of the host cells and therefore lead to a decrease in transduction. *In vivo* data of liver transduction, measured at week 2 postinfection, provided no significant difference between IOD AAV8 and POR AAV8 in terms of % GFP-positive hepatocytes, while slightly higher transduction was found by IOD AAV8 in terms of the GFP MFI signal (Figure 6(b)).

**Figure 7:**
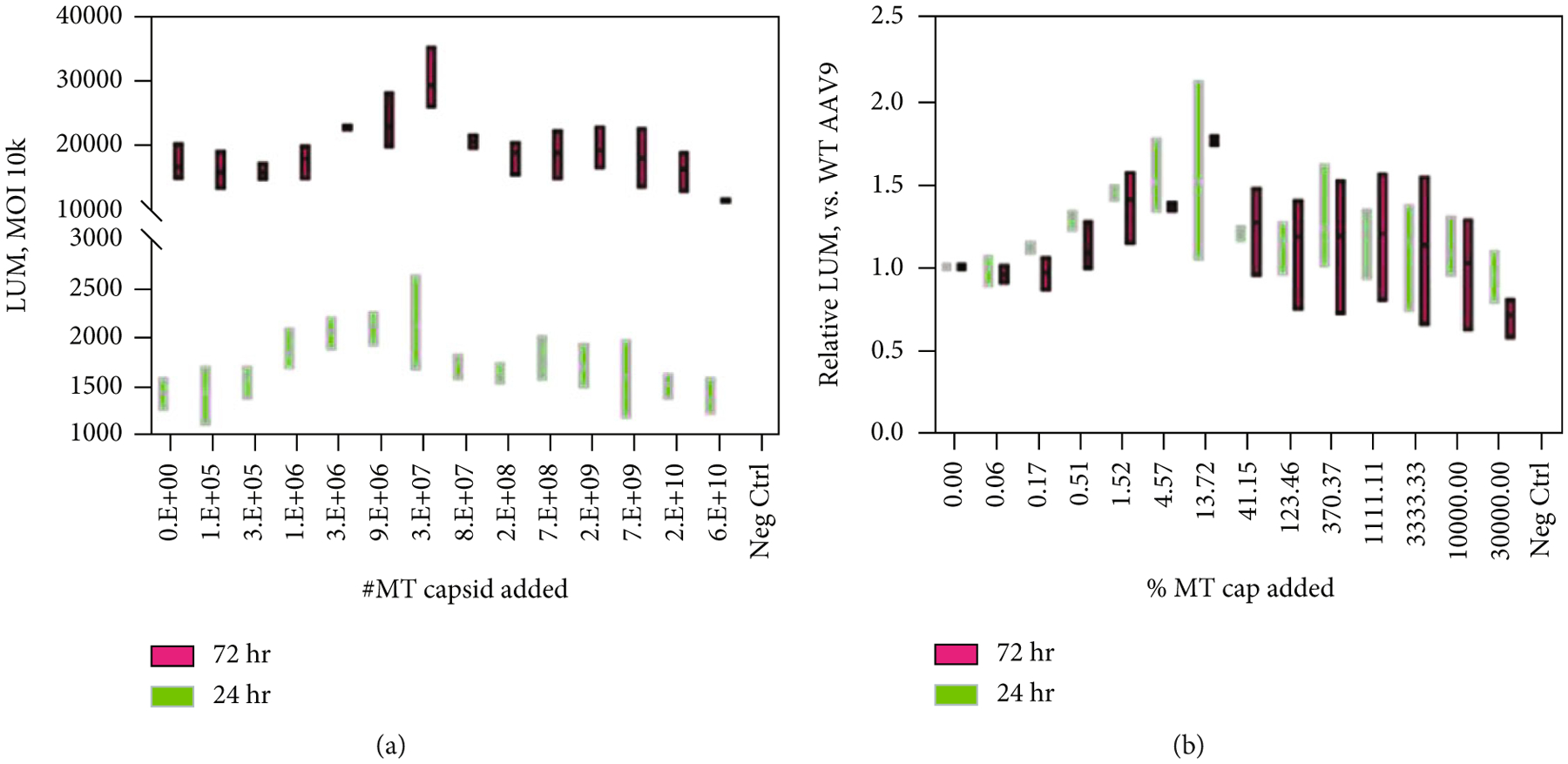
Transduction results of AAV9-CB-GLUC vs. empty capsids added on GM16095 cells. *N* = 3 wells/group. Luminometer readouts at 24 hrs (green) and 72 hrs (red) postinfection (a). Relative LUM readouts compared to WT AAV9 (b). MT cap = empty capsids.

As shown in Figure 7, a serial dilution of empty capsids of AAV9 added to the same MOI of AAV9-CB-GLUC led to variable transduction outcomes. The addition of empty capsids did not detrimentally affect the infectivity of AAV9 when that percentage of empty capsids was under ~41% (Figures 7(a) and 7(b)), as the transduction readout of luminescence was not lower. When the amount of empty capsids added exceeds 100%, the standard deviation becomes broader, leading to a difficult conclusion. This result shows that *in vitro*, transduction efficiency may vary depending on how much empty capsids are present.

**Figure 8:**
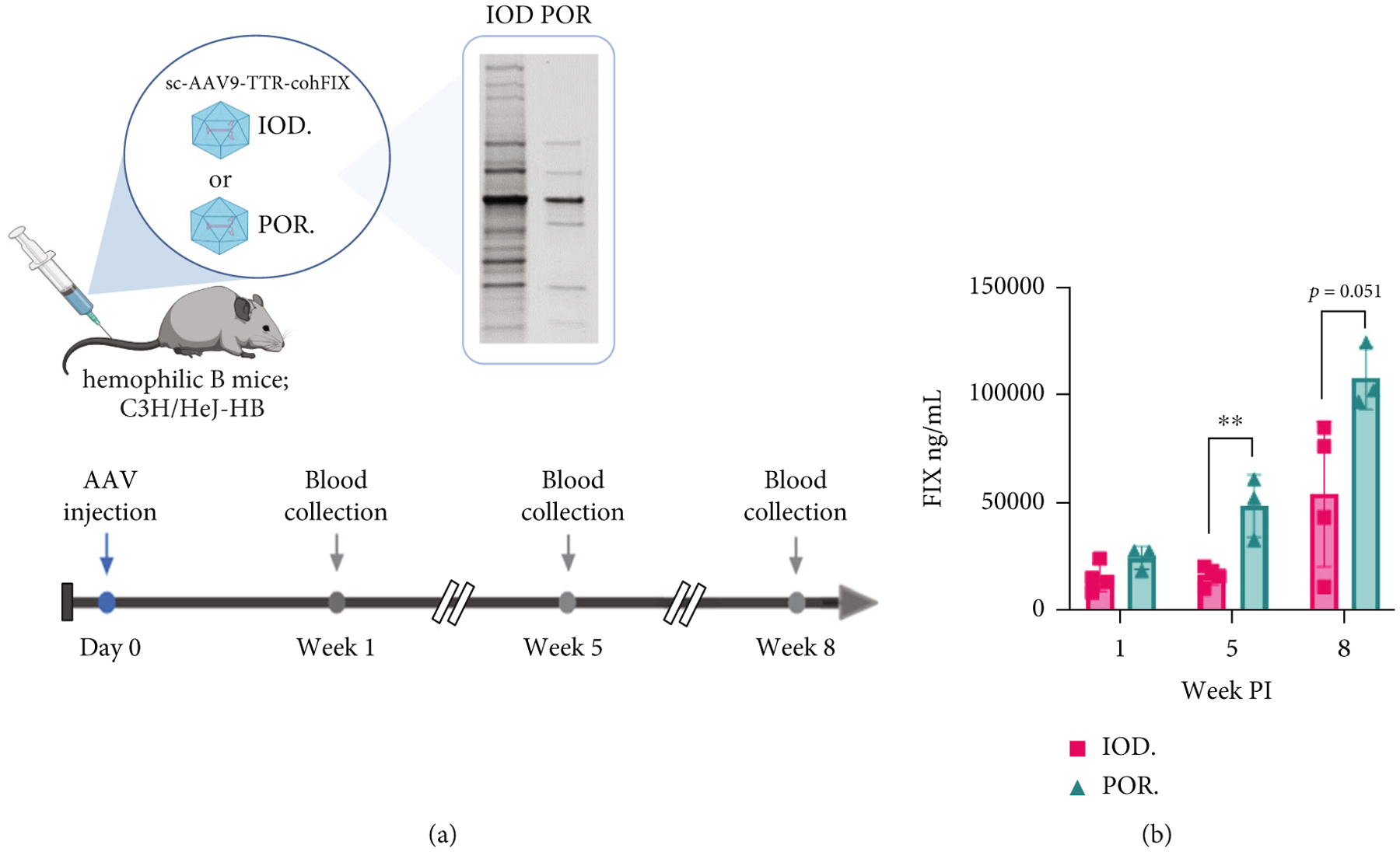
Comparison of systemic hFIX expression in hemophilia B mice. Study design with an inlet of SDS-PAGE for purity comparison (a). Plasma level of hFIX in ng/mL (b). Dose = 1 × 10^11^ vg/mouse; *n* = 3–4/group. No difference was found between the two purification methods found at week 1 PI, but weeks 5 and 8 showed a clearer difference in which POROS-AAVX purified AAV9 achieved better transduction efficacy compared to iodixanol purified AAV9. Two-tailed unpaired *t*-test, **p value < 0.01.

In the third batch of AAV, we produced AAV9-TTR-cohFIX, which is a codon-optimized construct encoding human factor IX (hFIX) for treating hemophilia B. The hemophilic B mouse model, C3H/HeJ-HB, was used to test the functionality of the differently purified vectors (Figure 8(a)). At weeks 1, 5, and 8 post-IV injections of the AAV, we collected blood for hFIX ELISA. The efficacy was measured based on the amount of circulating hFIX. While no significant difference was found at week 1 postinjection, POR AAV9 outperformed IOD AAV9 at week 5 and week 8 time points, yielding significantly ~2-fold higher hFIX levels (Figure 8(b)).

## 3. Discussion

The preparation and purification processes related to AAV production are essential for the sound interpretation of translational and preclinical investigations. As there are multiple methods to purify AAVs, it is imperative to research details related to how these processes affect different serotypes and batch preparations of AAVs. In this study, we report comprehensive comparison data from three self-complementary AAV serotype batches (AAV5, AAV8, and AAV9) prepared independently and purified by iodixanol gradient centrifugation (IOD) or affinity chromatography using the POROS^™^ CaptureSelect^™^ AAVX column (POR). These data include characterizations of purity, the relative empty capsid content (SDS-PAGE and transmission electron microscopy), titers of vg recovery, and plasmid backbone contaminations (qPCR). Our focus was to assess transduction efficiency *in vitro* and *in vivo* in WT mice plus a clinically relevant model of hemophilia B (C3H/HEJ-HB mice), as designed in Figure 1.

In this study, we employed a scalable vector production system utilizing a suspension of HEK293 cells that were cultured and maintained at a viable cell density and percentage of viability, as shown in Figure 2. Each batch of vectors was halved for the IOD or POR purification approach, followed by further characterization and transduction efficiency analyses. One standout advantage of affinity column purification is improved consistency in the high-level purity of AAV products, while iodixanol gradient centrifugation often yields more impurities like ferritin and other host proteins (Figures 3(a) and 3(b) and 4). In contrast, the iodixanol gradient method can be implemented independently of the capsid serotype, while affinity chromatography is reported to be effective for certain wild-type serotypes. AAV empty capsids have been described to vary in different vector production systems, can comprise up to 90% of total AAV particles generated, and even within one production system, empty capsid content can fluctuate highly between manufacturing batches [15]. One limitation of our study is that no absolute quantification of empty capsids per method of purification was calculated. Nevertheless, as we observed from the TEM images and SDS-PAGE gels, the relative empty capsids in each batch of AAV product purified by POR were always higher than those from IOD (Figures 3 and 4). Vector recovery (vg titer from qPCR) from both methods was comparable, as shown in Figure 3(c). Using a relative comparison of multiplexed qPCR, DNA impurities from plasmid backbones measured by ampicillin primers and probes were found to be ~6% by both purification methods (Figure 3(d), dark color bars). Pretreatment of DNase I prior to qPCR titering reduced the DNA impurity by about 1%, which indicated that some of the plasmid backbones were present outside of the capsid and ~5% were packaged by the vector. Other studies have reported that this backbone contamination could be a result of defective virions (including single ITR and snapback genome vectors) and can be minimized by having a transgene size close to the maximum packaging capacity of the rAAV (~5 kb) [16, 17].

*In vitro*, POR AAV vectors were found to transduce GM16095 human fibroblasts less efficiently than their counterpart IOD AAVs (Figures 5(a) and 6(a)). One hypothesis was that the higher ratio of empty capsids in POR AAV preparations competes with the full particles for receptor binding on host cells and leads to lower gene expression. To test this hypothesis, we generated AAV9-CB-GLUC vectors spiked with different amounts of empty capsids (Figure 7). To detrimentally affect transduction efficacy, a large number of empty particles were required (greater than 41% empty capsids added to lower the LUM readout). However, the *in vivo* data for each batch did not fully corroborate the *in vitro* results. As shown in Figure 5(b), POR AAV5 yielded slightly higher gene expression of % GFP-positive cells compared to IOD AAV5 in the mouse liver at week 2 postinjection, although no significant statistical difference was found due to a large standard deviation. In contrast, Figure 6(b) shows that POR AAV8 yielded slightly lower gene expression of % GFP-positive liver cells as compared to IOD AAV8 in the mouse liver at week 2 postinjection. These mixed *in vivo* results between the two batches of AAV5 and AAV8 could be from the end-point analysis at week 2 PI because the gene expression delivered by different serotypes of AAV might require different times to be fully expressed. The third batch of AAV9-packaged codon-optimized human factor IX (hFIX) was added to the study to assess functional differences in the preparation methods in a clinically relevant disease model for AAV gene therapy. As shown in Figure 8 using a hemophilia B mouse model, no significant difference in hFIX was found in the plasma between IOD and POR AAV9 at week 1 PI. However, at week 5 and week 8 PI, plasma hFIX delivered by POR AAV9 was significantly higher than those delivered by the IOD AAV9, suggesting that a higher purity vector preparation (other than empty capsids) may be beneficial for *in vivo* gene delivery. There are several possible contributions to these disparate results between *in vitro* and *in vivo* transduction including impurities or a variety of empty capsids in each batch since we did not normalize the empty capsid of each serotype for the *in vivo* experiments. Regardless, our data showed that vectors purified by affinity chromatography could successfully deliver the gene of interest in mice, if not better, compared to iodixanol gradient centrifugation, despite the higher content of empty capsids. In a previous study reported by Blessing et al. [18], intrastriatal injection in adult mice showed that AAV2/9 purified by the iodixanol gradient centrifugation method had significantly higher transduction than vector purified by the affinity POROS AAV column at week 4 postinjection. This could be due to the empty capsid content, batch-to-batch variation, or timing differences in their experiment compared to ours presented here.

While the iodixanol gradient can separate full and empty particles from one another, residual iodixanol in the final AAV product may pose a safety concern and may warrant additional purification steps. Pu et al. have reported an LCMS method for the detection of residual iodixanol [19]. As investigational AAV-based products continue to move through clinical development, more *in vivo* studies are needed to examine the impact of downstream processing of AAV vectors, particularly focusing on differences in the ratio of empty-to-full viral particles and impurities present in vector batches. Some clinical trials and preclinical studies intentionally mixed AAV empty capsids to act as decoys for neutralizing anti-AAV antibodies and therefore enhanced gene transfer [20]. In other cases, they were undesirable as they could elicit liver toxicity due to activation of CD8^+^ T cells and TLR2 [21–23]. Thus, studying how the systematic addition of empty particles impacts the immune response is important to identify a threshold of empty capsid content that maximizes both safety and transduction efficacy. Given the adverse side effects in multiple clinical trials of AAV-based drugs [24] and the less well-understood effects of impurities like empty capsids and residual plasmid DNA, regulatory agencies have proposed risk mitigation plans that include the manufacturing process of AAV to decrease the percentage of empty capsids in 2021 [25]. Many studies have proposed chromatography methods to separate full and empty capsids using ion-exchanged columns based on the slight difference in charge possessed by the nucleic acid content of the full virions [5, 26–28].

Any physiochemical and biological properties (full-to-empty ratio, purification method, formulation excipients, phenotypic variation, capsid serotype, infectivity per dose per route of administration, etc.) should be well documented, even though the effects of these on vector performance might not be fully understood. With more knowledge about AAVs being documented, studied, and disseminated, detailed bookkeeping and certification on process development would be a valuable tool for risk mitigation to ensure the safety and well-being of patients in the clinic.

## 4. Conclusion

Neither the IOD nor POR purification method was found to be superior overall. Each purification method was found to have its own advantages and disadvantages (summarized in Table 1). *In vitro* data show that IOD-purified vectors outperformed POR-purified ones. Empty capsids were found to decrease the infectivity of AAVs *in vitro* at high concentration. However, *in vivo* data show variable results from batch-to-batch of different AAVs with no consistent trend. Titer normalization, purity, and types of impurities in a final AAV product may play a big component in determining vector potency. In conclusion, iodixanol gradient centrifugation and affinity chromatography have their own advantages and limitations, and both are similarly capable of yielding infectious AAV vectors for research scale. For manufacturing on a large scale, additional processes are needed to minimize impurities in each batch for more consistent quality control of the final AAV product.

## 5. Materials and Methods

### 5.1. rAAV Production.

The suspension cell line HEK293F was used for rAAV vector production. The cells were cultured in viral production medium (Thermo, Cat#A4817901) in shaker flasks at 120 rpm at 37°C, 8% CO_2_. Cell viability was maintained at >95%, and transfection was performed when the cell density was at ~2–2.5E6 cells/mL using the standard triple-plasmid transfection method of pRep2CapX (X = serotype 5, 8, and 9), pHelper, and pITR-AAV (pds-CB-EGFP, CB-GLUC, or TTR-cohFIX) at an equal molar ratio, plus transfection agent Fectovir. For empty capsid production, no p-ITR-AAV was used. These cells were collected at 72 hr posttransfection. Centrifugation at 3000 g for 15 min was performed to separate the cells and media. The cells were resuspended in lysis buffer of 50 mM Tris-HCl (+0.15 M NaCl, pH 8.2), went through 3 cycles of freeze/thaw to release rAAV viruses, and centrifuged at 3000 g for 20 min to collect the supernatant. This supernatant was combined with the cell media and treated with DNase I (5 U/mL) for 1 hr at 37°C to digest contaminant DNAs and improve the quality of the crude lysate. The rAAV viruses in the crude were purified using either iodixanol gradient centrifugation or liquid chromatography with an affinity column (POROS AAVX CaptureSelect, Thermo #A36651).

### 5.2. rAAV Purifications.

*Iodixanol gradient ultracentrifugation* followed the Addgene protocol using Option #3, puncturing the QuickSeal tube slightly below the 60–40% interface (https://www.addgene.org/protocols/aav-purification-iodixanol-gradient-ultracentrifugation/).

An *immuno-affinity chromatography* detailed protocol was reported by Lam et al. [29].

Briefly, the POROS^™^ CaptureSelect^™^ AAVX column was used with an AKTA GO system controlled by UNI-CORN 7.6 software. The equilibration and wash buffer were PBS, pH 7.2. The secondary wash buffer was 18% ethanol and PBS+1 M NaCl. The elution buffer was 0.05 M citric acid+0.1 M glycine+0.1% p188 (pH 2.9). Eluted fractions were neutralized by 1 M Tris-HCl buffer (pH 8.2), and then the buffer was exchanged with PBS and concentrated down using centrifugal Vivaspin, 20 mL, 100 K MWCO.

### 5.3. SDS-PAGE.

Mini-PROTEAN TGX precast gel (7.5% polyacrylamide gel, Bio-Rad) was used. Samples plus Laemmli buffer containing 10% of *β*-mercaptoethanol were mixed and heated at 90°C for 5 min. The samples were cooled to room temperature and loaded into the gel lanes, together with a standard marker. The running buffer was 1 × Tris/glycine/SDS, Bio-Rad. The assembly was set and connected. The voltage was set to be constant at 200 V. The gel was set to electrophorese for 30 min. The gel was removed from the cassette and imaged according to the manufacturer’s protocol using a ChemiDoc MP Imaging System (Bio-Rad).

### 5.4. Transmission Electron Microscopy.

Purified AAV samples (3 *μ*L) were negative stained with NanoVan (3 *μ*L) on a nickel Formval/Carbon 300 mesh. The samples were air-dried before being imaged at 120 kV using a Tecnai Spirit equipped with an AMT CCD camera (Thermo Fisher).

### 5.5. In Vitro Cell Culture and Transduction Assays.

GM16095 human fibroblast cells were purchased from the Coriell Institute and grown in 10% FBS DMEM with 1% antibiotics at 37°C with 5% CO_2_. The cells were subcultured once they reached 90% confluency. For transduction assays, the cells were counted and seeded in a 96-well plate 24 hrs before transduction with rAAVs. Based on the AAV transgene of each experiment, the cells were trypsinized for flow cytometry to determine % GFP positive or measured for Gaussia luciferase activity (using the Pierce Gaussia Luciferase Glow Assay Kit, Thermo Fisher #16161, following the manufacturer’s protocol).

### 5.6. Animal In Vivo Experiments.

All mice were maintained in the laboratory animal resource center at Indiana University–Purdue University, Indianapolis (IUPUI). All animal experimental protocols were approved and performed as per the guidelines of Indiana University’s Institutional Bio-safety Committees (IBC) and Institutional Animal Care and Use Committee (IACUC). Each specific experiment (specified in each result figure) includes either WT BALB/c mice (purchased from the Jackson Lab) or hemophilia B C3H/HeJ-HB mice (in-house breed) [30]. All mice were treated with rAAVs (purified by either iodixanol centrifugation or affinity chromatography) at 8–10 weeks old; the negative control group was mock-injected with PBS. AAV dosage was 1E11 to 2E11 vg/mouse (see figure legends for details of each experiment). For the BALB/c mice, at sacrifice (week 2 postinjection), their livers were harvested for further flow cytometry analysis. For the C3H/HeJ-HB mice, plasma samples were collected by retroorbital eye bleed into 0.38% sodium citrate.

### 5.7. Flow Cytometry Analysis.

Mouse livers were processed on the day of harvest and measured for eGFP signal based on a previously described protocol [31] with a slightly modified digestion step as follows: a single lobe was digested in 7 mL of digestion media (0.2 mg/mL collagenase P, 5 U/mL DNase I, 1.5 U/mL dispase, and 1% FBS in RPMI 1640). Simple gating for unfixed, single cells from the liver was set for direct fluorescence of GFP+ cells from negative control GFP cells using the Attune NxT Flow Cytometer.

### 5.8. hFIX ELISA.

Enzyme-linked immunosorbent assay-(ELISA-) based measurements of human FIX from the mouse plasma were carried out as previously described [32].

### 5.9. Statistics.

GraphPad Prism 9.1.2 software was used to calculate all statistics. Unless otherwise stated, data are presented as means ± standard deviations.

## Figures and Tables

**Table 1:** Comparative properties of iodixanol gradient centrifugation versus immuno-affinity chromatography.

	Iodixanol gradient centrifugation	Immuno-affinity chromatography
Purity	Poorer	Better
Yield monitor	No	Yes (A280 nm)
AAV serotypes	Independent	Depend on column type
Time-consuming	Yes (more steps: PEG precipitation)	Faster + automatic system
Impurities	Residual iodixanol & large proteins	Small proteins
Empty capsids	Less	More
Scalability	No	Yes
GMP readiness	Poor	Good

## Data Availability

The datasets used and/or analyzed during the current study are available from the corresponding author on reasonable request.

## References

[1] Ozelo MC, Mahlangu J, Pasi KJ , “Valoctocogene roxaparvovec gene therapy for hemophilia a,” New England Journal of Medicine, vol. 386, no. 11, pp. 1013–1025, 2022. 35294811 10.1056/NEJMoa2113708

[2] Crossrates M, AveXis receives FDA approval for Zolgensma^®^, the first and only gene therapy for pediatric patients with spinal muscular atrophy (SMA), Crossrates M, 2019.

[3] Prado DA, Acosta-Acero M, and Maldonado RS, “Gene therapy beyond luxturna: a new horizon of the treatment for inherited retinal disease,” Current Opinion in Ophthalmology, vol. 31, no. 3, pp. 147–154, 2020. 32175942 10.1097/ICU.0000000000000660

[4] Naso MF, Tomkowicz B, Perry WL 3rd, and Strohl WR, “Adeno-associated virus (AAV) as a vector for gene therapy,” BioDrugs, vol. 31, no. 4, pp. 317–334, 2017. 28669112 10.1007/s40259-017-0234-5 PMC5548848

[5] El Andari J and Grimm D, “Production, processing, and characterization of synthetic AAV gene therapy vectors,” Biotechnology Journal, vol. 16, no. 1, 2021. 10.1002/biot.202000025 32975881

[6] Zhao H, Meisen WH, Wang S, and Lee KJ, “Process development of recombinant adeno-associated virus production platform results in high production yield and purity,” Human Gene Therapy, vol. 34, no. 1–2, pp. 56–67, 2023. 36401498 10.1089/hum.2022.153

[7] Toueille M, “Development of purification steps for several AAV serotypes using POROS^™^ CaptureSelect^™^ AAVX affinity chromatography,” Cell and Gene Therapy Insights, vol. 2, no. 1, 2016.

[8] Jacobson SG, Acland GM, Aguirre GD , “Safety of recombinant adeno-associated virus type 2-RPE65 vector delivered by ocular subretinal injection,” Molecular Therapy, vol. 13, no. 6, pp. 1074–1084, 2006. 16644289 10.1016/j.ymthe.2006.03.005

[9] Weleber RG, Pennesi ME, Wilson DJ , “Results at 2 years after gene therapy for RPE65-deficient Leber congenital amaurosis and severe early-childhood-onset retinal dystrophy,” Ophthalmology, vol. 123, no. 7, pp. 1606–1620, 2016. 27102010 10.1016/j.ophtha.2016.03.003

[10] Lowes LP, Alfano LN, Arnold WD , “Impact of age and motor function in a phase 1/2A study of infants with SMA type 1 receiving single-dose gene replacement therapy,” Pediatric Neurology, vol. 98, pp. 39–45, 2019. 31277975 10.1016/j.pediatrneurol.2019.05.005

[11] Foust KD, Wang X, McGovern VL , “Rescue of the spinal muscular atrophy phenotype in a mouse model by early postnatal delivery of SMN,” Nature Biotechnology, vol. 28, no. 3, pp. 271–274, 2010. 10.1038/nbt.1610 PMC2889698 20190738

[12] Rodrigues GA, Shalaev E, Karami TK, Cunningham J, Slater NKH, and Rivers HM, “Pharmaceutical development of AAV-based gene therapy products for the eye,” Pharmaceutical Research, vol. 36, no. 2, p. 29, 2018. 30591984 10.1007/s11095-018-2554-7 PMC6308217

[13] Administration UFaD, Chemistry, manufacturing, and control (CMC) information for human gene therapy investigational new drug applications (inds). 2020, Administration UFaD, 2021.

[14] Grieger JC, Soltys SM, and Samulski RJ, “Production of recombinant adeno-associated virus vectors using suspension HEK293 cells and continuous harvest of vector from the culture media for GMP FIX and FLT1 clinical vector,” Molecular Therapy, vol. 24, no. 2, pp. 287–297, 2016. 26437810 10.1038/mt.2015.187 PMC4817810

[15] Wright JF, “Product-related impurities in clinical-grade recombinant AAV vectors: characterization and risk assessment,” Biomedicines, vol. 2, no. 1, pp. 80–97, 2014. 28548061 10.3390/biomedicines2010080 PMC5423478

[16] Zhang J, Chrzanowski M, Frabutt DA , “Cryptic resolution sites in the vector plasmid lead to the heterogeneities in the rAAV vectors,” Journal of Medical Virology, vol. 95, no. 1, article e28433, 2023. 36571262 10.1002/jmv.28433 PMC10155192

[17] Zhang J, Guo P, Yu X , “Subgenomic particles in rAAV vectors result from DNA lesion/break and non- homologous end joining of vector genomes,” Molecular Therapy-Nucleic Acids, vol. 29, pp. 852–861, 2022. 36159586 10.1016/j.omtn.2022.08.027 PMC9463555

[18] Blessing D, Vachey G, Pythoud C , “Scalable production of AAV vectors in orbitally shaken HEK293 cells,” Molecular Therapy - Methods & Clinical Development, vol. 13, pp. 14–26, 2019. 30591923 10.1016/j.omtm.2018.11.004 PMC6305802

[19] Pu Y, Katz R, Chen Y , “Development and application of a liquid chromatography-mass spectrometry method for residual Iodixanol quantification in AAV-based gene therapy product development,” Human Gene Therapy, vol. 33, no. 1–2, pp. 103–108, 2022. 34376063 10.1089/hum.2021.136 PMC10112872

[20] Mingozzi F, Anguela XM, Pavani G , “Overcoming preexisting humoral immunity to AAV using capsid decoys,” Science Translational Medicine, vol. 5, no. 194, 2013. 10.1126/scitranslmed.3005795 PMC4095828 23863832

[21] Wright JF, “Codon modification and PAMPs in clinical AAV vectors: the tortoise or the hare?,” Molecular Therapy, vol. 28, no. 3, pp. 701–703, 2020. 32035026 10.1016/j.ymthe.2020.01.026 PMC7054817

[22] Hösel M, Broxtermann M, Janicki H , “Toll-like receptor 2–mediated innate immune response in human nonparenchymal liver cells toward adeno-associated viral vectors,” Hepatology, vol. 55, no. 1, pp. 287–297, 2012. 21898480 10.1002/hep.24625

[23] Mingozzi F, Maus MV, Hui DJ , “CD8(+) T-cell responses to adeno-associated virus capsid in humans,” Nature Medicine, vol. 13, no. 4, pp. 419–422, 2007. 10.1038/nm1549 17369837

[24] Paulk N, “Gene therapy: it is time to talk about High-dose AAV,” Genetic Engineering & Biotechnology News, vol. 40, no. 9, pp. 14–16, 2020.

[25] (FDA) UFaDA, Toxicity risks of adeno-associated virus (AAV) vectors for gene therapy (GT), Cellular, Tissue, and Gene Therapies Advisory Committee (CTGTAC) Meeting #70, 2021.

[26] Gagnon P, Goricar B, Mencin N , “Multiple-monitor HPLC assays for rapid process development, in-process monitoring, and validation of AAV production and purification,” Pharmaceutics, vol. 13, no. 1, p. 113, 2021. 33477351 10.3390/pharmaceutics13010113 PMC7830902

[27] Dickerson R, Argento C, Pieracci J, and Bakhshayeshi M, “Separating empty and full recombinant adeno-associated virus particles using isocratic anion exchange chromatography,” Biotechnology Journal, vol. 16, no. 1, article e2000015, 2021. 33002276 10.1002/biot.202000015

[28] Hejmowski AL, Boenning K, Huato J, Kavara A, and Schofield M, “Novel anion exchange membrane chromatography method for the separation of empty and full adeno-associated virus,” Biotechnology Journal, vol. 17, no. 2, article e2100219, 2022. 34921599 10.1002/biot.202100219

[29] Lam AK, Zhang J, Frabutt D , “Fast and high-throughput LC-MS characterization, and peptide mapping of engineered AAV capsids using LC-MS/MS,” Molecular Therapy - Methods & Clinical Development, vol. 27, pp. 185–194, 2022. 36284765 10.1016/j.omtm.2022.09.008 PMC9563341

[30] Cao O, Hoffman BE, Moghimi B , “Impact of the underlying mutation and the route of vector administration on immune responses to factor IX in gene therapy for hemophilia B,” Molecular Therapy, vol. 17, no. 10, pp. 1733–1742, 2009. 19603001 10.1038/mt.2009.159 PMC2835008

[31] Finlon JM, Burchill MA, and Tamburini BAJ, “Digestion of the murine liver for a flow cytometric analysis of lymphatic endothelial cells,” Journal of Visualized Experiments, vol. 143, no. 143, article e58621, 2019. 10.3791/58621 PMC6350926 30663671

[32] Kung S-H, Nathan Hagstrom J, Cass D , “Human factor IX corrects the bleeding diathesis of mice with hemophilia B,” Blood, The Journal of the American Society of Hematology, vol. 91, no. 3, pp. 784–790, 1998. 9446637

